# TIP-B1 promotes kidney clear cell carcinoma growth and metastasis via EGFR/AKT signaling

**DOI:** 10.18632/aging.102298

**Published:** 2019-09-27

**Authors:** Lei Yin, Shenglin Gao, Heng Shi, Keyi Wang, Huan Yang, Bo Peng

**Affiliations:** 1 Department of Urology, Shanghai Tenth People’s Hospital, School of Medicine in Tongji University, Shanghai, China; 2 Department of Urology, Changzhou No. 2 People’s Hospital, Nanjing Medical University, Changzhou, Jiangsu, China; 3 Department of Urology, Shanghai Tenth People’s Hospital, Nanjing Medical University, Nanjing, China; 4 Department of Urology, Tongji Hospital, Tongji Medical College, Huazhong University of Science and Technology, Wuhan, China

**Keywords:** TIP-B1, kidney clear cell carcinoma (KIRC), AKT, EGFR

## Abstract

Kidney clear cell carcinoma (KIRC) is the most prevalent kidney malignancy. Accumulating evidence shows that high expression of TIP-B1 correlates with development of tumor progression. However, the detailed functions of TIP-B1 in the KIRC remain to be further elucidated. Here, we firstly found TIP-B1 expression was significantly increased in KIRC compared with adjacent normal tissues. What’s more, higher expression of TIP-B1 were correlated with aggressive clinico-pathological characteristics. In vitro assay found TIP-B1 knockdown dramatically inhibited KIRC cells proliferation, migration and invasion. In vivo assay found down regulated TIP-B1 could suppress tumor growth and metastasis. Mechanism analysis indicated that TIP-B1 could bind EGFR and suppress EGFR degradation, then promoted EGF-induced AKT signaling. Together, TIP-B1 could be applied as an independent risk factor to predict KIRC progression and metastasis. Targeting TIP-B1 might be a new potential therapeutic strategy for KIRC treatment.

## INTRODUCTION

Renal cell carcinoma (RCC) is the most prevalent primary kidney malignancy and accounts for an estimated 90–95% of kidney cancer cases [1]. Unfortunately, approximately one-third of RCC patients found metastatic lesions at the time of initial diagnosis, and about half of the remaining patients eventually found metastatic lesions during postoperative follow-up [2]. Kidney clear cell carcinoma (KIRC) is the most prevalent subtype of RCC. Metastasis is also the leading cause of poor survival in KIRC patients [3], but the exact regulatory mechanisms are still unclear. Therefore, identifying prognostic biomarkers and therapeutic targets that contribute to the KIRC metastasis are extremely important.

Recently, with microarray or high-throughput sequencing, many novel genes are found to be highly expressed in tissue-specific tumor [4, 5]. Through GEO database and TCGA database, we found TIP-B1 is significantly upregulated in KIRC tumor. TIP-B1, a new gene which has been identified in 1999 [6], also known as SH3BGRL3, belong to SH3BGR family [7]. During the past years, the function of SH3BGR family is largely unknown. Extremely, little is known about the role of TIP-B1 in tumors. Wang et al found murine SH3BGRL (mSH3BGRL) strongly promoted tumor cell invasion and lung metastasis, but human SH3BGRL (hSH3BGRL) in turn suppressed tumorigenesis and metastasis [8]. Previous study found TIP-B1 was increasing in glioblastoma multiform [9]. More importantly, some studies reported TIP-B1 was detected in lung adenocarcinoma and bladder cancer patient urine [10, 11], which indicated TIP-B1 may play a key role in urothelial carcinoma. However, whether TIP-B1 could promote KIRC growth and metastasis still need to be elucidated.

In the present study, through comparing some public databases, we found TIP-B1 significantly increased in KIRC tumor tissues, especially in metastatic tumor. Besides, we found TIP-B1 expression was positively correlated with progression and recurrence in KIRC patients. Univariate and multivariate analyses indicated that TIP-B1 could be applied as an independent risk factor to predict KIRC progression and metastasis. Functionally, TIP-B1 knockdown suppressed KIRC growth and metastasis both in vitro and in vivo. Moreover, we demonstrated that TIP-B1 activated the EGFR/ AKT signaling pathway and enhanced the epithelial–mesenchymal transition (EMT). In summary, our study presented that TIP-B1 was a novel candidate for inhibiting KIRC growth and metastasis.

## RESULTS

### TIP-B1 is overexpressed in KIRC and has aggressive clinicopathological prediction trait

**Figure 1:**
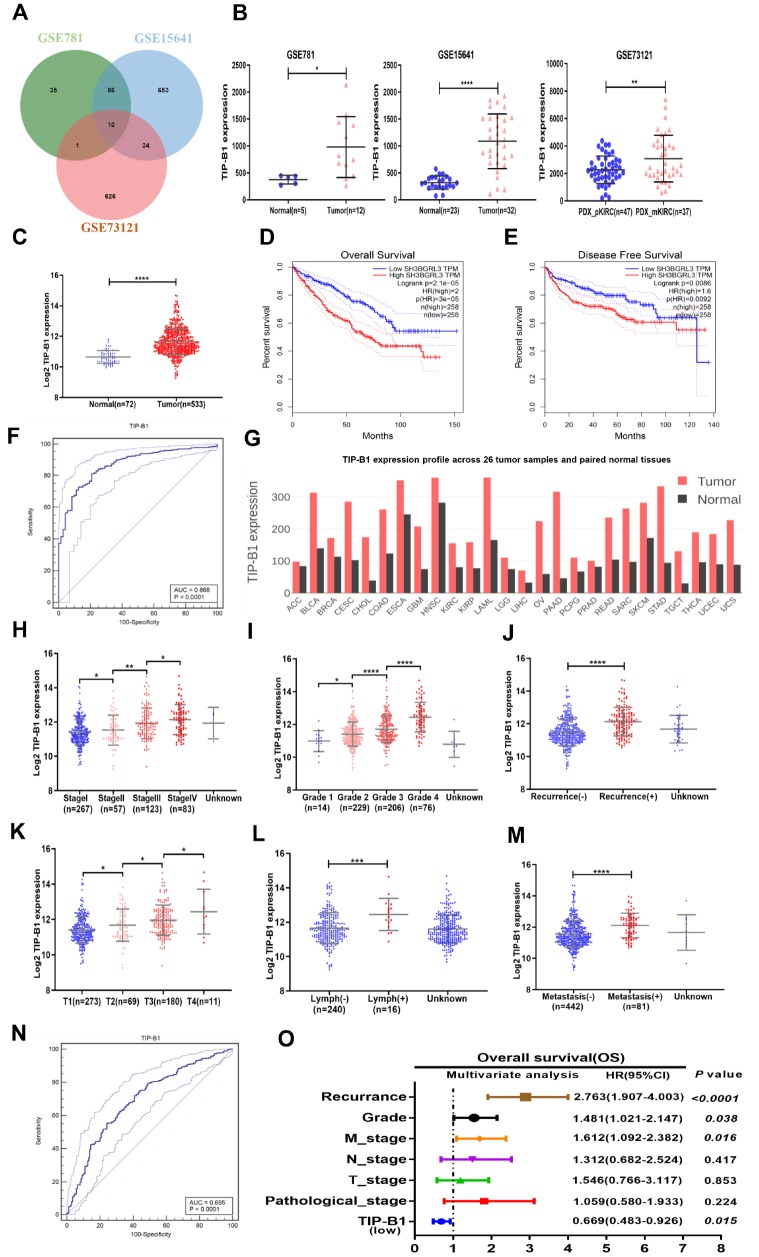
**Identify TIP-B1 is overexpressed in KIRC and predict aggressive clinicopathological traits.** (**A**) Venn diagram of differentially expressed genes in GSE781, GSE15641and GSE73121datasets. (**B**) The TIP-B1 in KIRC tumor samples and pair-matched tissues. Each dot represents one sample. (**C**) TCGA cohort analysis of the TIP-B1 in KIRC tumor samples and pair-matched normal tissues. (**D**–**E**) Overall survival (**D**) and disease-free survival (**E**) curve of KIRC patients with low and high TIP-B1 expression. (**F**) ROC curve of TIP-B1 between KIRC and normal tissues (**G**) TIP-B1 expression across 26 human tumor and paired tissues. (**H**–**M**) Relative expression levels of TIP-B1 in TCGA-KIRC subgroup: pathological stage (**H**) tumor grade (**I**) recurrence status (**J**) tumor stage (**K**) lymphatic invasion (**L**) metastasis status (**M**). (**N**) ROC analysis to assess the specificity and sensitivity of TIP-B1 to differentiate between high and low group in KIRC tumor group. (**O**) Multivariate cox regression analyses of TIP-B1 expression with overall survival in TCGA database. The HR are presented as the means (95% confidence interval). *p < 0.05, **p < 0.01, ***p < 0.001, ****p < 0.0001.

To identify potential molecules which may play key role in KIRC metastasis, we download three KIRC microarray and RNA sequencing data (GSE781, GSE15641, GSE73121) from GEO database. GSE781 contained 5 adjacent non-tumor tissue and 12 KIRC tumors. GSE15641 contained 23 adjacent non-tumor tissue and 32 KIRC tumors. GSE73121 contained 47 primary KIRC and 37 metastatic KIRC patient-derived xenografts (PDX) tissues. The detailed gene expression data were listed Supplementary Table 6. After merging those 3 datasets, 10 significantly upregulated genes both in tumor and metastasis tissues were identified (Figure 1A, Supplementary Table 6). We further validated those 10 genes in TCGA-KIRC cohort and found only TIP-B1 expression was significantly correlative in receiver operating characteristics (ROC) curves associating with KIRC genesis (Figure 1F, Supplementary Figure 1), which suggested TIP-B1 might be a good indicator for KIRC. As shown in Figure 1B, TIP-B1 was dramatically increased both in tumor tissues and metastatic PDX models. Then we analyzed the expression of TIP-B1 in TCGA-KIRC cohort and found that TIP-B1 expression was also significantly upregulated in tumor tissues when compared with adjacent non-tumor kidney tissues (Figure 1C). Besides, upregulation of KIRC mRNA level were also confirmed by the Oncomine database (https://www.oncomine.org/) (Supplementary Figure 2). In addition, GEPIA webtools (http://gepia.cancer-pku.cn/), Kaplan–Meier overall survival (OS) analysis and disease free survival (DFS) analysis indicated that KIRC patients with the higher level of TIP-B1 had dramatically shorter OS (Figure 1D) and DFS (Figure 1E) than those with the lower level of TIP-B1, which indicated TIP-B1 might paly central role in KIRC progression. Moreover, tumor (red) samples had significantly higher TIP-B1 expression when compared to paired normal (black) samples across 26 human tumor types (Figure 1G) from GEPIA webtools, and poorer prognosis in many human cancers, including esophageal carcinoma, head-neck carcinoma, liver carcinoma, pancreatic ductal adenocarcinoma, and rectum adenocarcinoma (Supplementary Figure 3), which further suggested that TIP-B1 might play an oncogenic role in various human cancer types.

To future identify changes of TIP-B1 expression during KIRC development, we analyzed the TCGA-KIRC dataset subgroups and found TIP-B1 expression was significantly correlated with several clinicopathological characteristics. For example, with the progression of pathological stage (Figure 1H), tumor grade (Figure 1I) and tumor stage (Figure 1K), the TIP-B1 expression significantly increased step by step. The Oncomine database also confirmed those phenomenon (Supplementary Figure 4).

Furthermore, we also analyzed the 5-year overall survival rate of each stage and grade, and results indicated high expression of TIP-B1 in each stage and grade also have worse OS rate (Supplementary Figure 5). Besides, higher TIP-B1 expression was also strongly correlated with easier tumor recurrence (Figure 1J), lymphatic invasion (Figure 1L) and metastasis (Figure 1M). Furthermore, we used ROC analysis to calculate the optimal cut-off value (11.767) of the expression of TIP-B1 with KIRC patients survival state, and revealed that TIP-B1 might serve as a good indicator for predicting KIRC progression (Figure 1N, Supplementary Figure 6 and Supplementary Table 1). Univariate and multivariable logistic regression models were performed to analyze the correlation of TIP-B1 levels with overall survival of KIRC patients. Patient characteristics were provided in Supplementary Table 2. Both univariate analysis and multivariate analysis (Figure 1O, Supplementary Table 3) indicated that high TIP-B1 expression level was an independent risk factor for worse overall survival, together with tumor recurrence, grade and metastasis.

In conclusion, the above data from GEO and TCGA human clinical samples demonstrated that higher expression of TIP-B1 was associated with the advanced tumor grade and stage. More importantly, higher expression of TIP-B1 was associated with tumor recurrence and metastasis and could be used as an independent prognostic marker for KIRC patients.

### TIP-B1 is frequently upregulated in KIRC patients and correlates with poor prognosis

**Figure 2:**
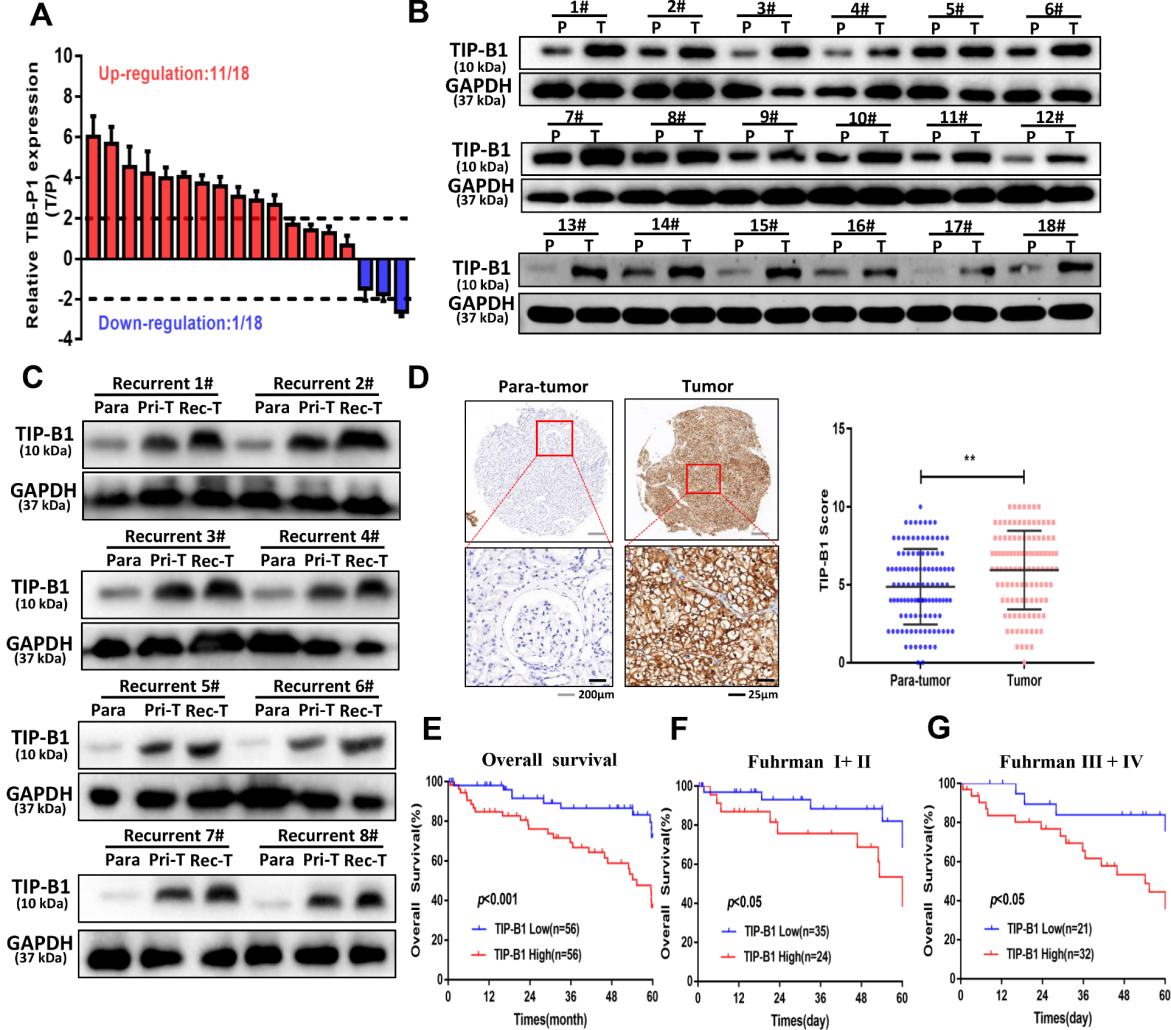
**TIP-B1 is frequently upregulated in KIRC patients and correlates with poor prognosis.** (**A**) RT-PCR analysis of TIP-B1 expression levels in 18 KIRC tissues and paired non-tumor kidney tissues. (**B**) Western blot analysis of TIP-B1 expression levels in 18 KIRC tissues and paired non-tumor kidney tissues. (**C**) Western blot analysis of TIP-B1expression levels in matched para-tumor, primary-tumor and recurrent tumor tissues from the same case. (**D**) Representative TMA images showing NR1B2 staining, and comparison of the IHC score between tumor and non-tumor kidney tissues. (**E**) Kaplan–Meier analysis of TMA patients with low versus high expression levels of TIP-B1. (**F**–**G**) Kaplan-Meier survival curve of TIP-B1 high-expressing and low-expressing patients was compared in Fuhrman stages I−II (**F**) and Fuhrman stages III−IV (**G**) subgroups.

Because TCGA dataset results were RNA-seq data, we wanted to demonstrate TIP-B1 expression both in RNA level and protein level. Therefore, we firstly collected 18 pairs of KIRC tissues and adjacent normal tissues, and detected the expression of TIP-B1 by RT-PCR and WB assay. The RT-PCR results demonstrated that TIP-B1 expression was significantly upregulated in KIRC tissues compared with adjacent normal tissues (Figure 2A) and WB assays also confirmed the RT-PCR findings (Figure 2B). Next, we focused on the relationship between TIP-B1 and KIRC prognosis. 8 recurrent patients samples were collected. WB assay showed that the protein level of TIP-B1 in paracancerous tissues, primary tumors and recurrent samples increased gradually (Figure 2C), which confirmed the TCGA cohort. Moreover, IHC staining from 112 KIRC patients tissue microarray (TMA) samples showed that KIRC tissues expressed higher protein level of TIP-B1 than adjacent kidney tissues (Figure 2D).

Then we calculated the expression of TIP-B1 and selected the median expression point as the cutoff criterion and divided those patients into high TIP-B1 group and low TIP-B1 group. As shown in Table 1, TIP-B1 expression significantly correlated with tumor size (P=0.033), Fuhrman grade (P=0.037), tumor stage (P=0.031) and metastasis (P=0.019) in KIRC. Overall survival analysis indicated that KIRC patients with high expression of TIP-B1 had significantly lower 5-year overall survival rates (Figure 2E), the same results were also confirmed by the Human Protein Atlas website (https://www.proteinatlas.org/) (Supplementary Figure 7). what’s more, higher expression of TIP-B1 significantly correlated with worse OS rate both in early Fuhrman stage (Figure 2F) and advanced Fuhrman stage (Figure 2G). To further study whether the dysregulation of TIP-B1 correlated with the OS rate of KIRC patients, we then performed Kaplan–Meier method and Cox proportional hazard model. As shown in Table 2, the up-regulation of TIP-B1 significantly correlated with worse overall survival in TMA patients, suggesting TIP-B1 might be a useful prognosis predictor for KIRC patients.

**Table 1:** Correlation between clinic-pathological parameters of patients enrolled.

**Clinical characteristics**	**No.of patients (n = 112)**	**TIP-B1 low (n=56)**	**TIP-B1 high (n=56)**	***P* value ^a^**
Age (years)				0.703
≤60	48	25	23	
>60	64	31	33	
Gender				0.001*
Male	61	22	39	
Female	51	34	17	
Tumor size				0.033*
≤4cm	69	40	29	
>4cm	43	16	27	
Laterality				**0.449**
Left	54	25	29	
Right	58	31	27	
Fuhrman grade				0.037*
G1-2	59	35	24	
G3-4	53	21	32	
T stage				0.031*
I-II	71	41	30	
III-VI	41	15	26	
Metastasis				0.019*
No	94	52	42	
Yes	18	4	14	

* statistically significant (*p* <0.05)

^a^ *p* value from Chi-square test

**Table 2:** Univariate and multivariate cox proportional regression analysis with overall survival.

	**Univariate analysis**			**Multivariate analysis**	
	**HR (95%CI)**	***P* value**		**HR (95%CI)**	***P* value**
**Age (years)**					
≤60	1.000	0.004*		1.000	0.007*
>60	3.191(1.458-6.986)			2.924(1.332-6.419)	
**TIP-B1**					
Low	1.000	0.002*		1.000	0.016*
High	3.161(1.529-6.356)			2.502(1.183-5.924)	
**Gender**					
Female	1.000	0.875		NA	
Male	0.949(0.495-1.821)				
**Tumor size**					
≤4cm	1.000	<0.0001*		1.000	0.546
>4cm	3.348(1.702-6.587)			1.983(0.215-18.263)	
**Laterality**					
Left	1.000	0.658		NA	
Right	1.158(0.606-2.211)				
**Fuhrman grade**					
G1-2	1.000	0.445		NA	
G3-4	1.289(0.672-2.471)				
**T stage**					
I-II	1.000	0.001*		1.000	0.906
III-VI	3.182(1.636-6.189)			1.132(0.145-8.817)	
**Metastasis**					
No	1.000	<0.0001*		1.000	0.219
Yes	3.757(1.928-7.322)			2.924(1.332-6.419)	

CI: confidence interval, HR: hazard ratio

* statistically significant (p <0.05)

*p* value from Cox regression analyses

Collectively, from public database to our TMA cohort, from RNA level to protein level, our data indicated that high level of TIP-B1 might play a critical role in KIRC progression and metastasis.

### TIP-B1 promotes proliferation, migration and invasion of KIRC cells

**Figure 3:**
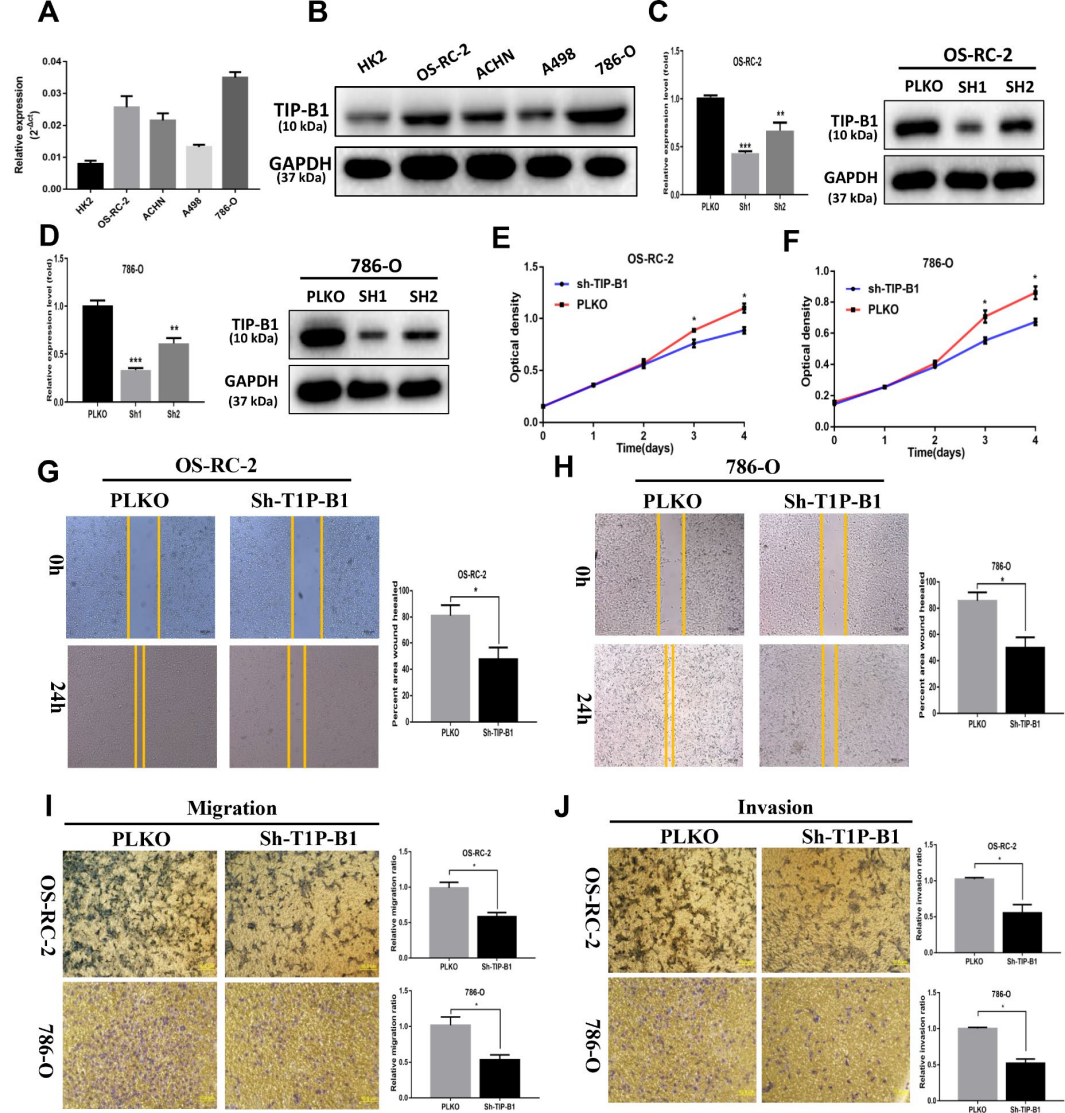
**TIP-B1 promotes proliferation, migration and invasion of KIRC cells.** (**A**) mRNA level of TIP-B1 in different KIRC cell lines and normal HK2 cell line. (**B**) protein level of TIP-B1 in different KIRC cell lines and normal HK2 cell line. (**C**–**D**) Efficiencies of TIP-B1 knockdown in OS-RC-2 cells (**C**) and 786-O cells (**D**) were validated by RT-PCR (left) and western blot(right) assays. (**E**–**F**) Cell proliferation was analyzed by CCK8 assay in OS-RC-2 cells (**E**) and 786-O cells (**F**). (**G**–**H**) Wound-healing assay after TIP-B1 knockdown in OSRC-2 (**G**) and 786-O (**H**) cells when compared to that of pLKO control cells. (**I**–**J**). Transwell migration (**I**) and invasion (**J**) assay after TIP-B1 knockdown in OSRC-2 (**I**) and 786-O (**J**) cells when compared to that of control cells.

In order to investigate the function of TIP-B1 in KIRC cells, we firstly detected TIP-B1 expression in human immortalized proximal tubule epithelial cell line HK-2 and KIRC cell lines 786-O, ACHN, OS-RC-2 and A498. The expression of TIP-B1 was significantly increased in KIRC cell lines when compared with immortalized adult human kidney cell HK2 both in RNA level (Figure 3A) and protein level (Figure 3B). Then we knocked down TIP-B1 by shRNA (sh-TIP-B1) in OS-RC-2 (Figure 3C) and 786-O (Figure 3D) cells which have a higher endogenous TIP-B1 expression, and chose the higher efficiency shRNA1 for further investigation. Functionally, cells with sh-TIP-B1 exhibited significantly decreased proliferation potential both in OS-RC-2 (Figure 3E) and 786-O (Figure 3F) compared with controls according to CCK-8 assay. Besides, cells with sh-TIP-B1 performed less migratory capability in OS-RC-2 (Figure 3G) and 786-O (Figure 3H) cells using wound-healing assay. Similarly, using the transwell migration and matrigel-coated invasion assay, we found that knocking down TIP-B1 in OS-RC-2 and 786-O cells decreased cell migration (Figure 3I) and invasion (Figure 3J) abilities compared to the vector control (pLKO) group.

In conclusion, results from in vitro assays demonstrated that TIP-B1 plays key role in regulating KIRC cell proliferation and a high level of TIP-B1 could increase KIRC cell migration and invasion abilities.

### TIP-B1 knockdown inhibits KIRC tumor growth and metastasis

**Figure 4:**
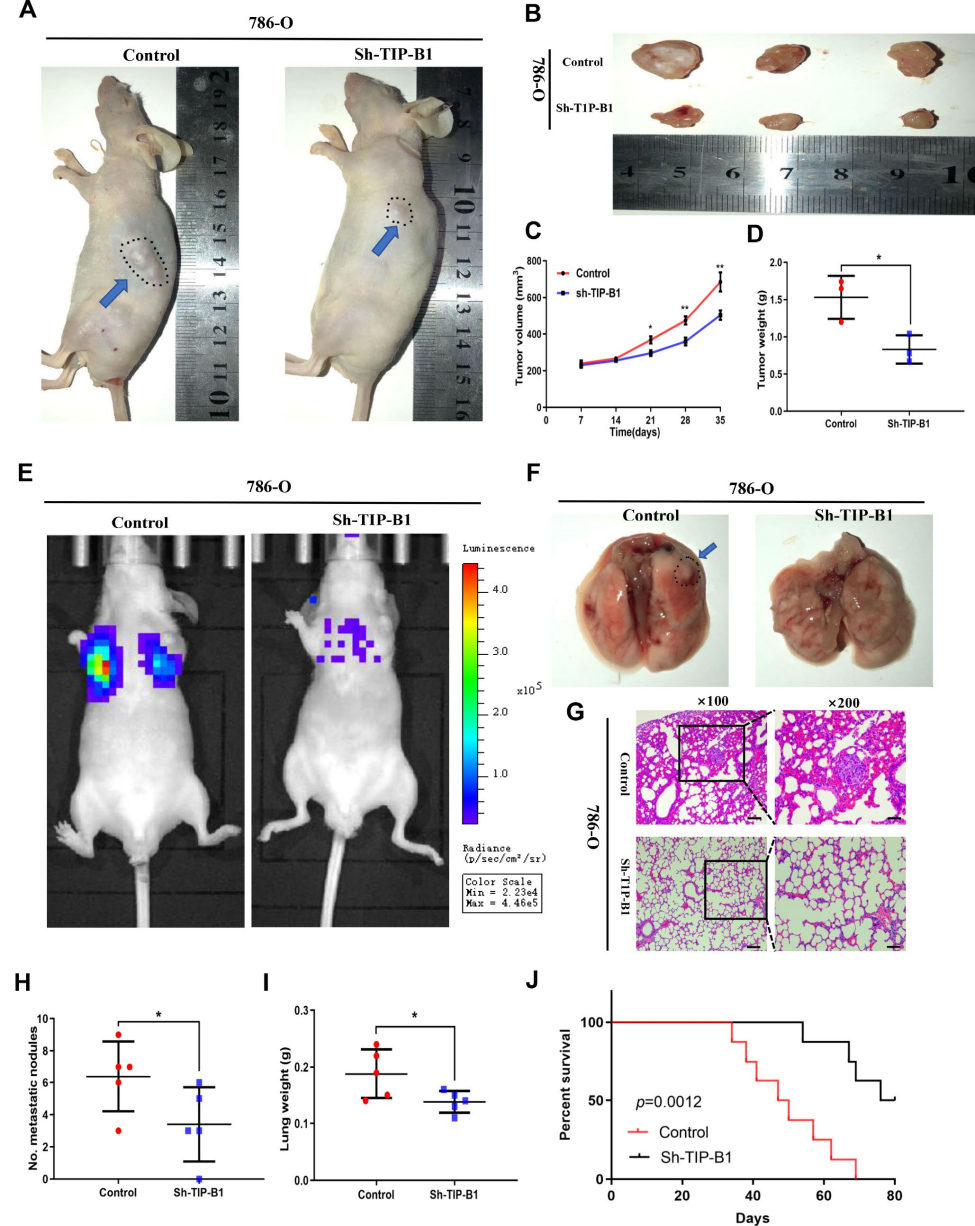
**TIP-B1 knockdown inhibits KIRC tumor growth and metastasis.** (**A**) Representative images of xenografts (arrows) were taken 5 weeks after injection. (**B**) The gross of tumors in sh-TIP-B1 and control groups. (**C**–**D**) Analysis of tumor volume (**C**) and weight (**D**) of xenograft tumors. (**E**) Representative images of metastasis by an in vivo bioluminescence imaging system. (**F**) Macroscopic appearance of lung metastatic nodule(arrows). (**G**) HE images of pulmonary micrometastases. (**H**) the number of pulmonary metastasis were compared. (**I**) Weights of the lung were compared. (**J**) Mouse survival curves.

To further confirm TIP-B1 function in vivo, 786-O cell line transfected with sh-TIP-B1 were subcutaneously implanted into nude mice. As expected, the tumor volume of sh-TIP-B1 group was much smaller than that of control group at 5 weeks (Figure 4A and 4B). Besides, the linear curve also recorded that knockdown TIP-B1 dramatically inhibited the growth (Figure 4C) and average weight (Figure 4D) of tumors in nude mice.

Next, we injected 786-O sh-TIP-B1 cells into tail vein of nude mice to simulate tumor metastasis. IVIS image showed that luciferase signal strength and area of sh-TIP-B1 group was significantly lower than control group (Figure 4E). Besides, the volume of micro-metastatic nodules markedly decreased in sh-TIP-B1 group (Figure 4F). HE analysis indicated the number and volume of pulmonary metastatic nodules were significantly decreased in sh-TIP-B1 group compared with control group (Figure 4G and 4H). In addition, the average lung weight in sh-TIP-B1 group was also dramatically lower than in control group (Figure 4I). More importantly, the mice injected with 786-O sh-TIP-B1 cells had significantly higher survival rates than control group (Figure 4J).

Taken together, our data demonstrated that inhibiting TIP-B1 could modulate the aggressive and metastatic abilities of KIRC in vivo.

### TIP-B1 triggers epithelial-mesenchymal transition by activating the AKT pathway

**Figure 5:**
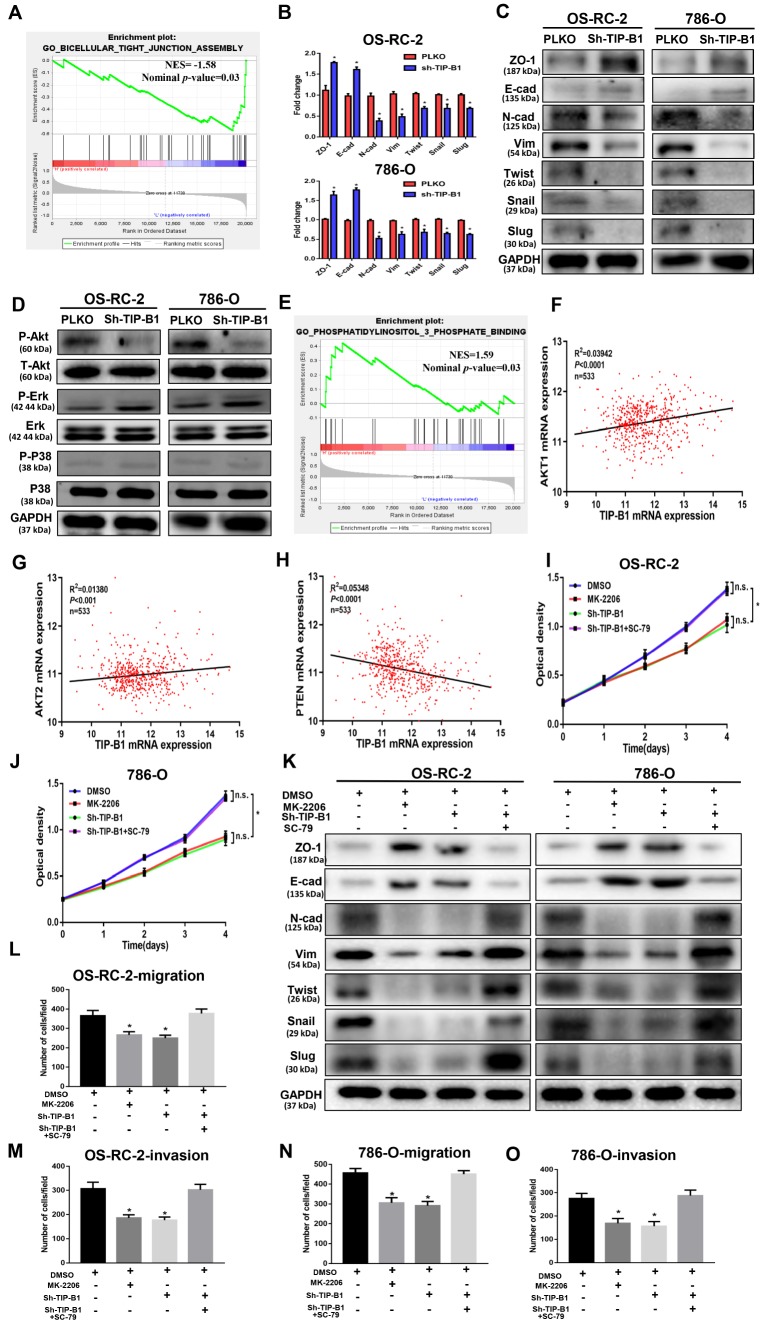
**TIP-B1 trigger epithelial-mesenchymal transition by activating the AKT pathway.** (**A**) GSEA analyses detected the tight junction markers were enriched in the TIP-B1 low group. (**B**–**C**) Expressions of EMT-related markers in KIRC cells with TIP-B1 downregulation by RT-PCR (**B**) and WB (**C**) assays. (**D**) Western blot assays detect candidate signal pathway. (**E**) GSEA analyses detected the AKT signal were enriched in TIP-B1 high group. (**F**–**H**) Correlation analysis between TIP-B1 and AKT1 (**F**), AKT2 (**G**), PTEN (**H**) in TCGA database. (**I**–**J**) Proliferation in the in OSRC-2 (**I**) and 786-O (**J**) cell lines was evaluated by CCK-8 assay. (**K**) Expressions of EMT-related markers in the indicated KIRC cells were detected by western blot assay. (**L**-**O**) Transwell migration (**L**, **N**) and invasion (**M**, **O**) assay was evaluated in indicated groups.

To explore the underlying molecular mechanisms of TIP-B1 in KIRC, we chose the TCGA-KIRC dataset and used median expression of TIP-B1 as the cutoff criterion and divided TCGA-KIRC dataset into high TIP-B1 group and low TIP-B1 group. Then we used the Gene set enrichment (GSEA) analysis to compare the different biological processes between the TIP-B1 high group and low group. Interestingly, GSEA analysis revealed that the tight junction function, an important part of the epithelial-mesenchymal transition (EMT) process, was significantly decreased in TIP-B1 high group (Figure 5A). It is well known that KIRC is mostly derived from renal tubular epithelial cells, and EMT is a key factor in the invasion and metastasis of epithelial-derived tumors [12, 13]. So firstly we tested EMT related markers. RT-PCR (Figure 5B) and WB (Figure 5C) results found that down regulated TIP-B1 had significantly increased the expression of epithelial related genes ZO-1, E-cadherin, and dramatically decreased mesenchymal related markers N-cadherin, vimentin, twist, snail and slug.

To elucidate the signal pathway that TIP-B1 plays role in, we detected AKT, p38(MAPK) and ERK, which were thought to be associated with KIRC deterioration and metastasis [14–16], and confirmed the phosphorylation of AKT molecules was clearly inactivated in both OS-RC-2 and 786-O sh-TIP-B1 cell lines, but ERK and p38 exhibited no changes (Figure 5D). Besides, the GSEA indicated phosphatidylinositol 3 phosphate binding genes enriched in TIP-B1 high group (Figure 5E), and TCGA correlation analysis also showed that TIP-B1 expression was positively correlated with AKT1 (Figure 5F), AKT2 (Figure 5G), and negatively with PTEN (Figure 5H), further demonstrating that TIP-B1 played a key role in the AKT pathway. In order to examine the effects of TIP-B1 on KIRC cell activities through AKT pathway, we treated KIRC cell with MK-2206 (a highly selective inhibitor of pan-AKT) or SC-79 (AKT phosphorylation activator) and divided cells into four groups (DMSO, MK-2206, sh-TIP-B1, sh-TIP-B1+SC-79). The CCK-8 assay showed both AKT inhibition and TIP-B1 knockdown could hindered KIRC cells proliferation, whereas AKT activation could attenuate the inhibitory effects of TIP-B1 knockdown on OS-RC-2 (Figure 5I) and 786-O (Figure 5J) cells. Besides, the increase of epithelial gene ZO-1, E-cadherin, and the decrease of mesenchymal gene N-cadherin, vimentin, twist, snail and slug caused by TIP-B1 knockdown could be rescued by AKT activation through WB assays in KIRC cells (Figure 5K). The rescue experiments of transwell assay also confirmed AKT inhibition could suppressed KIRC cells migration and invasion capacities, whereas AKT activation could release the inhibitory effects of TIP-B1 knockdown on KIRC cells (Figure 5L–5O, Supplementary Figure 8).

Together, our data suggested that TIP-B1 could trigger KIRC cells epithelial-mesenchymal transition by activating the AKT signaling pathway.

### TIP-B1/EGFR axis modulates AKT signaling in KIRC

**Figure 6:**
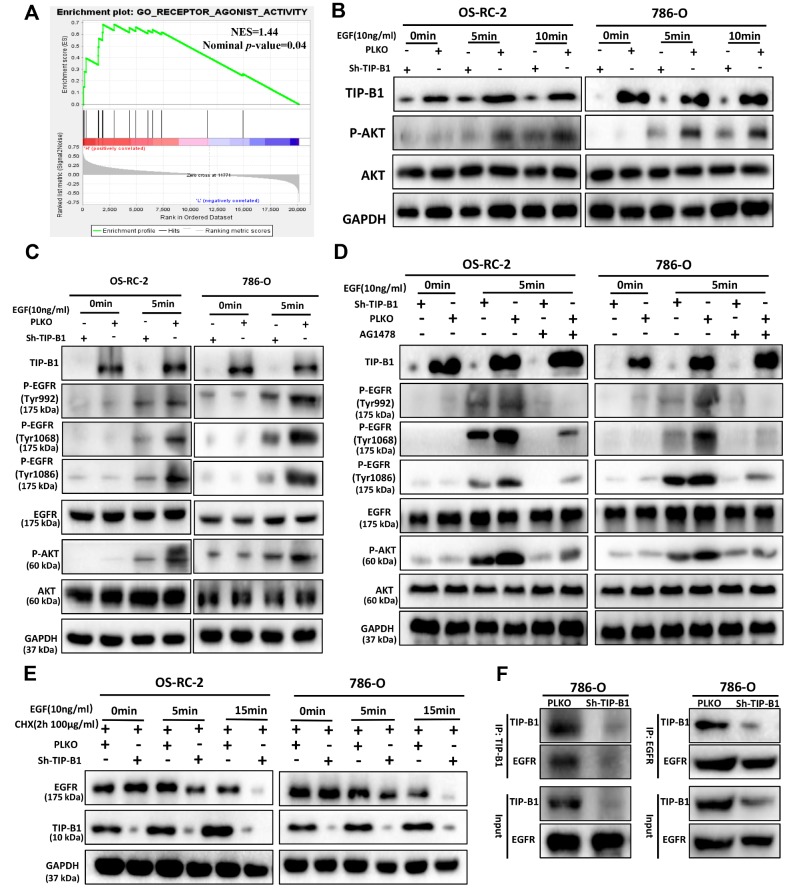
**TIP-B1/EGFR axis modulates AKT signaling in KIRC.** (**A**) GSEA analyses detected the receptor agonist activity were enriched in TIP-B1 high group. (**B**) Western blot assays detect the phosphorylation levels of AKT in OS-RC-2 (left) or 786-O cells (right) treated with EGF. (**C**) Western blot assays detect the phosphorylation levels of AKT and EGFR treated with EGF. (**D**) Western blot assays detect the phosphorylation levels of AKT and EGFR treated with EGF and EGFR specific inhibitor (AG1478). (**E**) Western blot assays detect EGFR with cycloheximide (**F**) Coimmunoprecipitation by TIP-B1(left) and EGFR (right) in786-O cells.

To investigate how TIP-B1 induces AKT signaling, we used GSEA analysis and found GO_RECEPTOR_ AGONIST_ACTIVITY was significantly enriched in TIP-B1 high group (Figure 6A), which means TIP-B1 interacts with some receptors to effect a change in the activity of those receptors. Previous studies have confirmed that activation of AKT signaling pathway is primarily through the interaction of receptor tyrosine kinases (RTKs) with their specific ligands, such as EGFR, which play important role in urological related tumors [17, 18]. So we tested whether TIP-B1 affected AKT signaling by interacting with EGFR. Interesting, knockdown TIP-B1 significantly inhibited AKT activation triggered by EGF stimulation compared to the control group (Figure 6B). More importantly, the phosphorylation levels of the three residues in EGFR (Tyr992, Tyr1068 and Tyr1086) were significantly reduced after downregulation of TIP-B1 (Figure 6C). Moreover, under the stimulation of EGFR-specific tyrosine kinase inhibitor (AG1478), the inhibitory effect of knockdown TIP-B1 on EGFR and AKT phosphorylation was furtherly enhanced, demonstrating that TIP-B1 might play a key role in EGFR mediated activation of AKT (Figure 6D).

To further investigate whether TIP-B1 knockdown could affect EGFR protein degradation, we detected the protein level of EGFR under EGF stimulation. The stability of EGFR protein was evaluated by cycloheximide (CHX, 100μg/ml), an inhibitor of protein synthesis, treatment for indicated times. Our data of WB analysis revealed that TIP-B1 knockdown could dramatically enhanced the degradation of EGFR (Figure 6E). Moreover, we sought to detect the physical interactions between TIP-B1 and EGFR by co-immunoprecipitation assays, and results revealed that the EGFR successfully co-immunoprecipitated TIP-B1 (Figure 6F).

Together, these results strongly suggested TIP-B1 might promote KIRC cells progression and metastasis by binding EGFR and suppressing EGFR degradation, then promoting EGF-induced AKT signaling.

## DISCUSSION

During the past years, the treatment of Kidney clear cell carcinoma (KIRC) has changed greatly, the 5-year disease free survival rate for patients with stage I and II has increased to 80–95% [19]. But for patients with stage III, the 5-year survival rate was 60% [20], and unfortunately, stage IV patients has only less than 10% of 5-year disease free survival rate [21]. The main cause of poor prognosis in KIRC patients with advanced stage is tumor metastasis. However, the molecular mechanism of KIRC progression and metastasis remains unclear.

TIP-B1, which is also named SH3BGRL3, is widely expressed and encodes for a highly conserved protein [22]. The crystal structure of TIP-B1 has been confirmed in recent research [23], but the function of TIP-B1 is still largely unknown. Recently, TIP-B1 was identified up-regulated in glioblastoma and papillary thyroid carcinoma versus normal tissues via GeLC-MS/MS technique [9, 24]. Moreover, Wang et al reported TIP-B1 could be a urine biomarker in lung adenocarcinoma and found TIP-B1 were more highly expressed in tumor compared to non-tumor tissues [10]. By comparing three public datasets (GSE781, GSE15641, GSE73121), we also found that TIP-B1 was significantly highly expressed in KIRC tumor tissues, especially in recurrence and metastasis groups. However, there is still limited knowledge about TIP-B1 correlation with progression of KIRC.

In order to study the function of TIP-B1 in KIRC, we firstly analyzed TCGA data of KIRC, and found TIP-B1 was significantly upregulated in tumor group than in adjacent non-tumor tissues. What’s more, both overall survival and disease-free survival analysis showed that KIRC patients with high levels of TIP-B1 had dramatically shorter OS and DFS than those with low levels of TIP-B1. Besides, GEPIA webtools also indicated TIP-B1 expression was significantly overexpressed and induced poor prognosis in many cancers, which furtherly suggested that TIP-B1 might play an oncogenic role in various human cancer types. In addition, our TMA samples also demonstrated a significant up-regulation of TIP-B1 protein levels in primary KIRC, especially in recurrent samples when compared to adjacent normal tissues. Taken together, multivariate analysis indicated that TIP-B1 could be used as an independent prognostic marker for KIRC patients both in RNA level and protein level.

It is well known that KIRC is mostly derived from renal tubular epithelial cells [25], and epithelial-mesenchymal transition (EMT) is a key step in the invasion and metastasis of epithelial-derived tumors [26–28]. Our functional experiments showed that reducing TIP-B1 could inhibit KIRC tumor cell proliferation, migration and invasion. Combined with the single gene GESA analysis which indicated TIP-B1 might be a key molecule in EMT, we supposed TIP-B1 might promote KIRC progression and metastasis by regulating EMT. Consistently, the WB assay found silent TIP-B1 lead to the alteration of EMT related markers. The subcutaneous tumor and lung metastasis model results also confirmed that down-regulation of TIP-B1 inhibited the growth and metastasis of mouse KIRC tumors.

There is no doubt that AKT activation plays an important role in the pathogenesis or progression of various tumors [29–31]. Many articles have reported that activation of AKT is significantly associated with higher KIRC tumor grade and tumor metastasis [32, 33]. In our current study, we found that inhibition of TIP-B1 expression could significantly attenuate the AKT signaling pathway, which was also confirmed by the GSEA and correlation analysis from TCGA database. Previous studies reported AKT activation promoted EMT, which in turn promoted distant metastasis of breast and lung cancer [34, 35]. In our study, we also identified that AKT signaling pathway played a key role in TIP-B1-induced KIRC cells EMT process. In addition, activation of AKT signaling is primarily mediated by interactions between receptor tyrosine kinases (RTKs) and their specific ligands, such as EGFR [36, 37]. Besides, EGFR has been reported to be associated with majority of solid tumors [38], and shows a positive correlation with the clinical histopathological characteristics in KIRC patients [39]. Interestingly, our data suggested that TIP-B1 has physical integration with EGFR and effectively prevented degradation of EGFR, then promoted EGF-induced AKT signaling.

**Figure 7:**
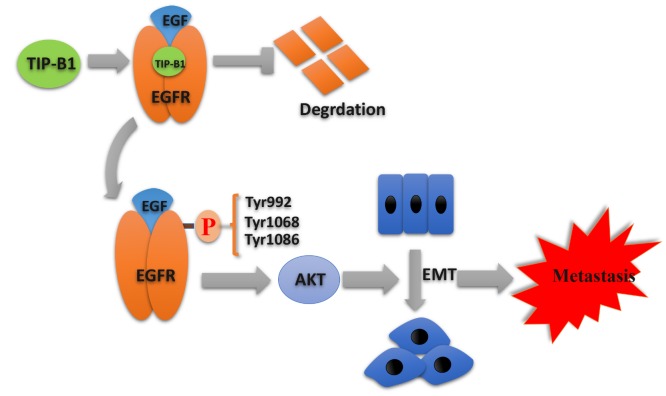
**Schematic depiction of the mechanisms underlying TIP-B1 mediated EGFR/AKT signaling to promote EMT and metastasis in KIRC.**

In summary, our research indicated a novel oncogene that TIP-B1 promoted KIRC growth and metastasis through EGFR/AKT signaling (Figure 7). Furthermore,

TIP-B1 could be used as an independent prognostic marker for KIRC patients who have undergone surgery, which not only provide a novel therapeutic strategy for future KIRC management, but also provide a potential target for cancer prevention and treatment.

## MATERIALS AND METHODS

### Human clinical samples

KIRC tumor samples and adjacent normal tissues were collected from the Department of Urology, Shanghai Tenth People’s Hospital, Tongji University. There were three groups were enrolled in this study. Briefly speaking, in group 1, fresh tumor tissues and adjacent non-tumor tissues from 18 KIRC patients were collected for RT-PCR and Western blot (WB) assays. In group 2, eight recurrent KIRC adjacent non-tumor, primary tumor and recurrent tumor tissues were collected for further WB assays. In group 3, 112 KIRC para-tumor and tumor specimens from 2008 to 2012 were collected for tissue microarrays (TMA) establishment.

### GEO database, TCGA database, ROC and GSEA analysis

Three public KIRC gene microarray profiling data (GSE781, GSE15641, GSE73121) were obtained from GEO database. GEO2R and R language were used to preprocess those three RNA sequencing and microarray data. The TIP-B1 RNA sequence data of KIRC were obtained from TCGA database. The ROC analyses were used to MedCalc software to calculate the sensitivities, specificities and accuracies at best cut-off point. As for single gene Gene set enrichment (GSEA) analysis, we used GSEA v3.0 to perform, and the median expression level of TIP-B1 was used as the cutoff criterion.

### Cell culture and lentiviral vector construction

The human immortalized proximal tubule epithelial cell line HK-2 and KIRC cell lines 786-O, ACHN, OS-RC-2 and A498 were obtained from Cell Bank of the Chinese Academy of Sciences. The HK-2 cells were cultured in Keratinocyte Serum Free Medium (K-SFM, Gibco, USA). The KIRC cell lines were cultured in Dulbecco’s Modified Eagle’s Medium (DMEM, Gibco, USA). All cells were supplement with 10% Fetal Bovine Serum (FBS, Gibco, USA). As for lentiviral vector construction, the pLKO/pLKO.1-shTIP-B1 plasmids, pMD2G envelope plasmid, psPAX2 packaging plasmid were transfected into HEK-293 cells using the standard calcium chloride transfection method. After incubating for 48 or 72 h, the supernatant medium was filtered and frozen in −80 °C for later use.

### CCK8, wound-healing assay and transwell assay

The CCK8 assay were conducted to calculate cell proliferation. Briefly speaking, cells were seeded in 200μL of fresh medium into 96-well plates (1000 cells/well) for various lengths of time at 37 °C in 5% CO2. For the wound-healing migration assay, a wound was made by scraping a 200-uL plastic yellow pipette tip along the bottom of the 6-well plate and observed and photographed at 0 h and 24 h after wounding. As for transwell migration and invasion (using matrigel) assay, 5×10^4^ cells/well in 200ul serum-free medium were added into the upper chambers, 600ul contained 10% FBS medium was added to the lower chambers. After 24 hours of incubation, the KIRC cells on the upper surface were clean up with cotton swab, and the lower surface were fixed with 4% paraformaldehyde, then stained by 0.1% crystal violet.

### Quantitative real-time PCR assay

Total RNAs were extracted using Trizol reagent (Invitrogen, CA). The cDNA were synthesized with Reverse Transcriptase Kit (Invitrogen, CA). The real-time PCR reactions were as follows: 5 min at 95°C, 40 cycles of 95°C for 10 s, then 60°C for 60 s. The relative expression were calculated using the ΔΔCt method. The primers sequence were listed in Supplementary Table 4.

### Western blot assay

Cells were lysed in RIPA lysis buffer (Beyotime, China) containing proteinase and phosphatase inhibitors on ice, and the concentration of protein was calculated through BCA method. Protein extraction (50μg) were seeded on SDS-polyacrylamide gel, then transferred to polyvinylidene fluoride (PVDF) membrane (Millipore, Billerica, MA). The PVDF membranes were incubated with primary antibodies overnight at 4 °C on a rocking table, and then incubated with the secondary antibodies for 1 hour at room temperature. The antibodies and reagents were listed in Supplementary Table 5.

### Tissue microarray and immunohistochemistry staining

The tissue microarray (TMA) construction has been previously described in detail [40]. Briefly speaking, 0.6 mm diameter tissue cylinders were punched from representative tissue area of each donor tissue block then brought into one paraffin block. Patients’ demographic and clinic-pathological were retrieved from our clinical database. Immunohistochemistry (IHC) experiments were carried out as described previously [41]. In general, the clinical human samples were incubated with the primary antibodies in 3% BSA in PBS overnight at 4 °C, followed by appropriate secondary antibodies. A semiquantitative scoring criterion which the staining index (values 0–10) were independently evaluated by three experienced pathologists.

### Animal studies

The animal protocols were approved by the Institutional Animal Use and Care Committee of Tongji University. All BALB/c nude mice (6 weeks) were purchased from the SLAC Laboratory Animal Company (Shanghai, China). As for xenografts group, 1×10^6^/100ul 786-O cells were inoculated subcutaneously into nude mice. The tumor volume of each mouse was measured every 7 days, and tumor weight were calculated after 5 weeks when mice were killed. In the pulmonary metastatic group, to evaluate the potential of the cells to metastasize to the lungs, ten male nude mice were randomized into 2 groups (n=5 each), and 1×10^6^/100ul cells were injected into the tail vein. The metastases were detected using the Lumina II IVIS system (Perkinelmer, USA). Those mice were also sacrificed after 5 weeks and weighed lung weight, then using H&E staining to analyze the metastatic lung foci. As for metastatic survival group, the same amount of cells injected into the tail vein, and the mouse survival was monitored in a survival analysis.

### Statistical analysis

Statistical analyses were using SPSS 23.0 software (IBM, USA). Continuous variables were presented as mean± SD. Two groups were analyzed by student t-test and multiple groups were used one way analysis of variance (ANOVA). The optimal cut-off value of the TIP-B1 expression was calculated by a ROC curve analysis through MedCalc software (MedCalc, Korea). Survival analysis were calculated using the Kaplan–Meier method, and evaluated by a log-rank test. The Cox proportional hazard model was used to determine the influencing factors based on the variables selected by univariate analysis. p < 0.05 was considered statistically significant. The detailed results were *p < 0.05, **p < 0.01, ***p < 0.001 and ****p < 0.0001.

## Supplementary Material

Supplementary Figures

Supplementary Table 1

Supplementary Tables 2-5

Supplementary Table 6
