# Supplementary material for: TIP-B1 promotes kidney clear cell carcinoma growth and metastasis via EGFR/AKT signaling
Source: Aging (Albany NY). 2019 Sep 27;11(18):7914–37. doi: 10.18632/aging.102298 (PMC6782011; doi:10.18632/aging.102298)
Supplement: Supplementary Figures [file aging-11-102298-s003.pdf]

SUPPLEMENTARY FIGURES

Sample size	605
Positive group [a]	533 (88.10%)
Negative group [b]	72 (11.90%)

[a] 0_normal = 1
[b] 0_normal = 0

Disease prevalence (%)	unknown

Area under the ROC curve (AUC)

Area under the ROC curve (AUC)	0.868
Standard Error [a]	0.0192
95% Confidence interval [b]	0.838 to 0.894
z statistic	19.169
Significance level P (Area=0.5)	<0.0001

[a] DeLong et al., 1988
[b] Binomial exact

Youden index

Youden index J	0.6022
Associated criterion	>10.9543
Sensitivity	81.05
Specificity	79.17

Supplementary Figure 1. The detailed process of ROC calculation with KIRC happened or not.

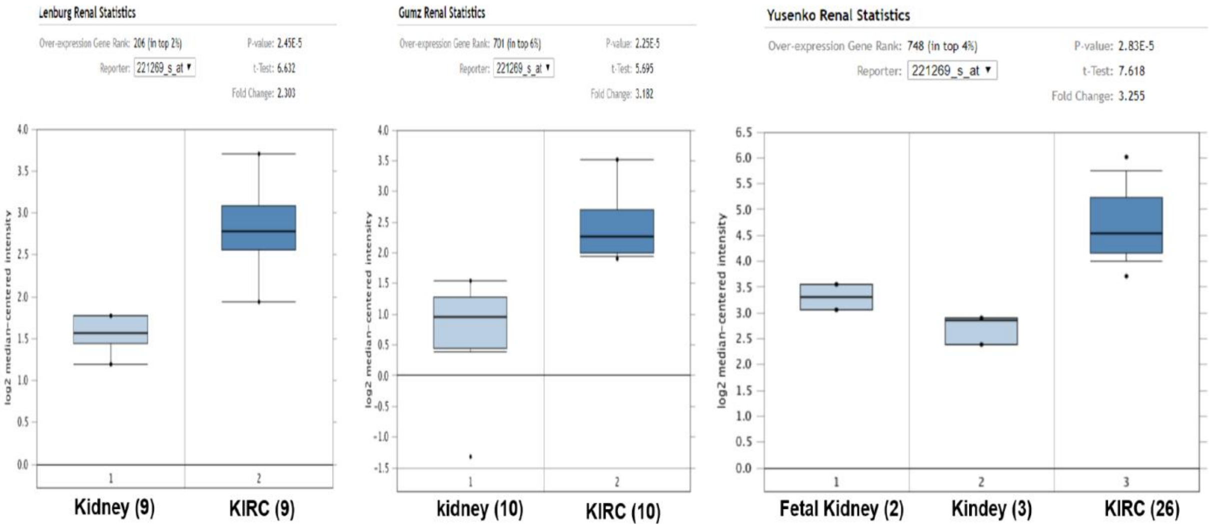

Supplementary Figure 2. Three data-group from Oncomine database indicate TIP-B1 upregulated in KIRC tumor.

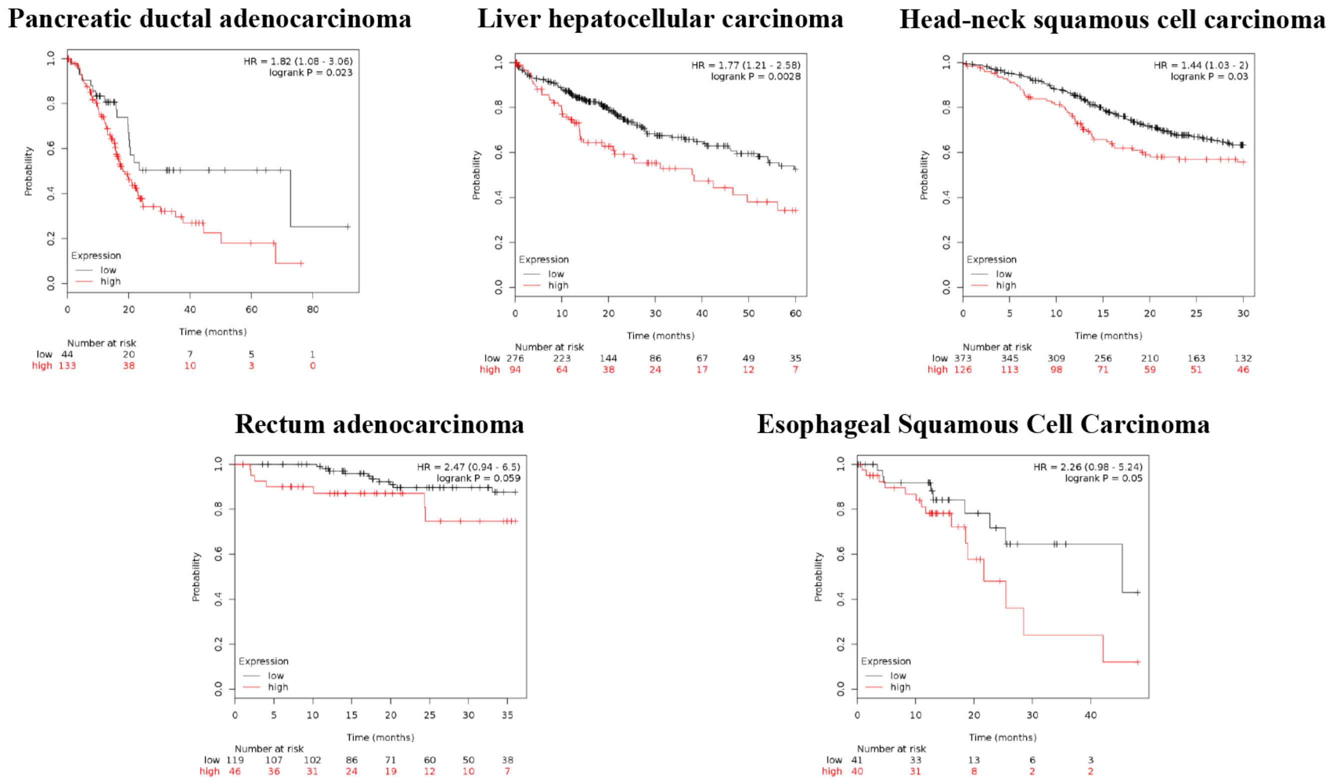

Supplementary Figure 3. High TIP-B1 indicate poor prognosis in many human cancers.

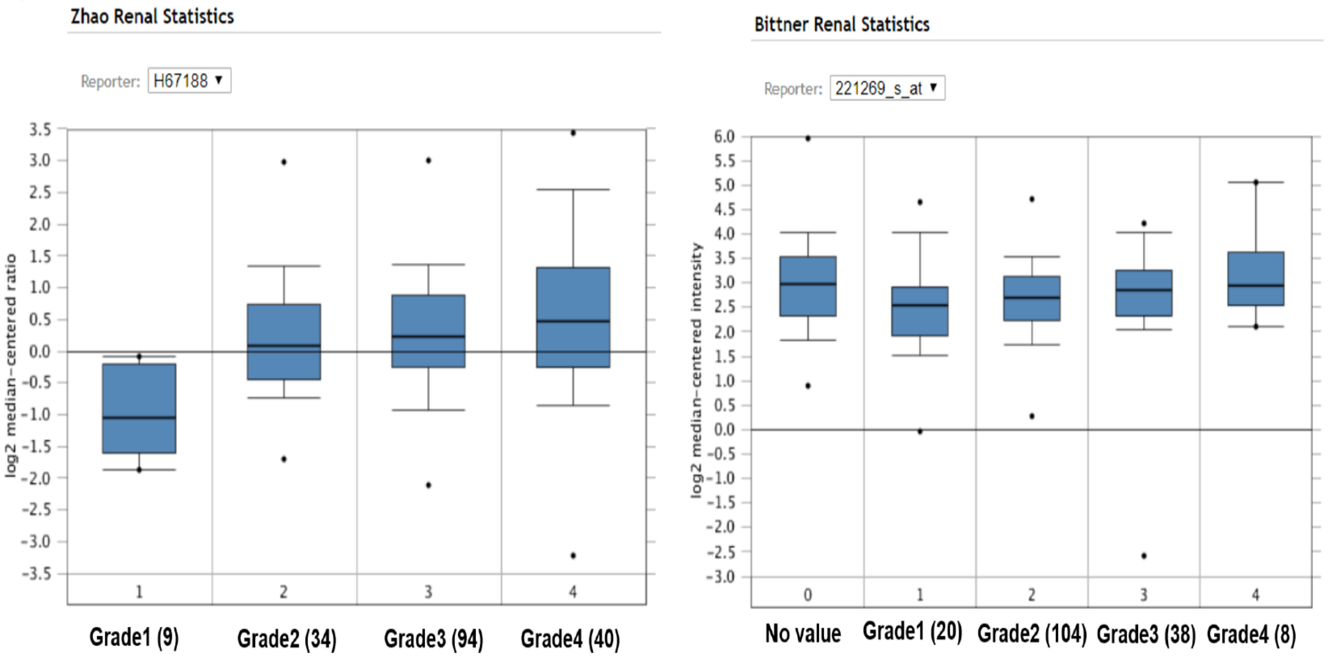

Supplementary Figure 4. The TIP-B1 expression significantly increased step by step in Oncomine database.

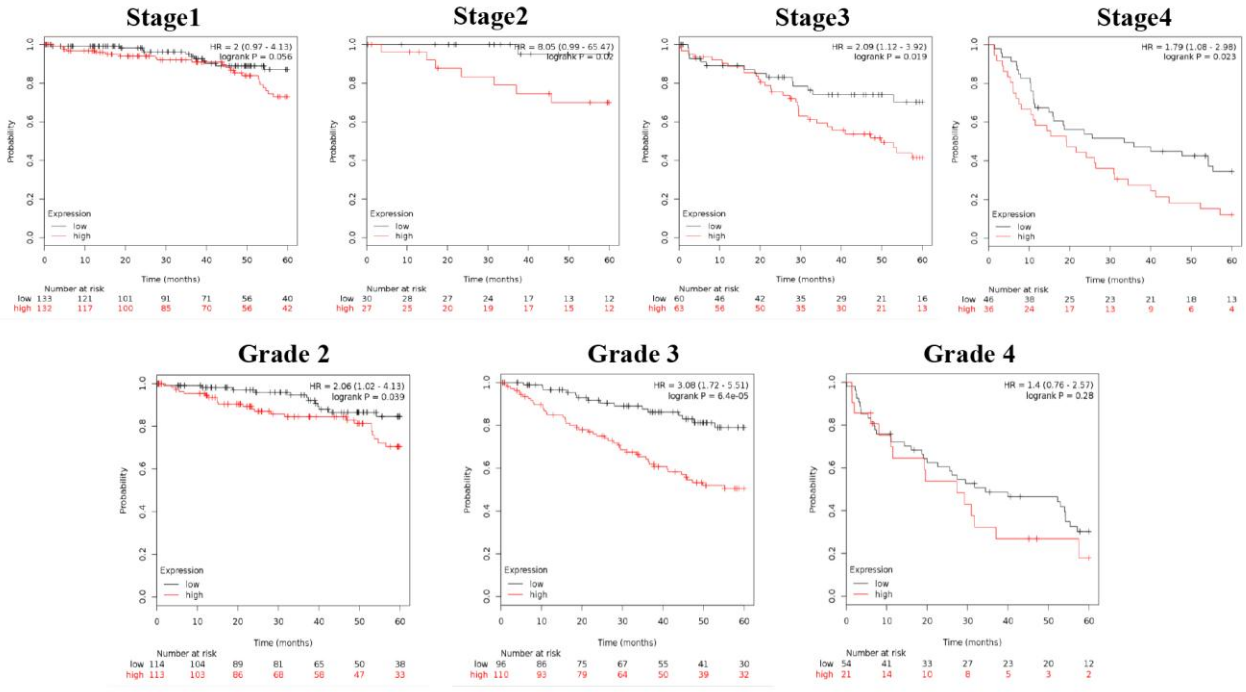

Supplementary Figure 5. High expression of TIP-B1 in each stage and grade have worse OS rate.

Sample size	533
Positive group [a]	359 (67.35%)
Negative group [b]	174 (32.65%)

[a] diagnosis = 1
[b] diagnosis = 0

Disease prevalence (%)	unknown

Area under the ROC curve (AUC)

Area under the ROC curve (AUC)	0.695
Standard Error [a]	0.0244
95% Confidence interval [b]	0.654 to 0.734
z statistic	7.990
Significance level P (Area=0.5)	<0.0001

[a] DeLong et al., 1988
[b] Binomial exact

Youden index

Youden index J	0.3106
Associated criterion	≤11.767
Sensitivity	71.87
Specificity	59.20

Supplementary Figure 6. ROC analysis to calculate the optimal cut-off value of TIP-B1 associated with KIRC patients survival or not.

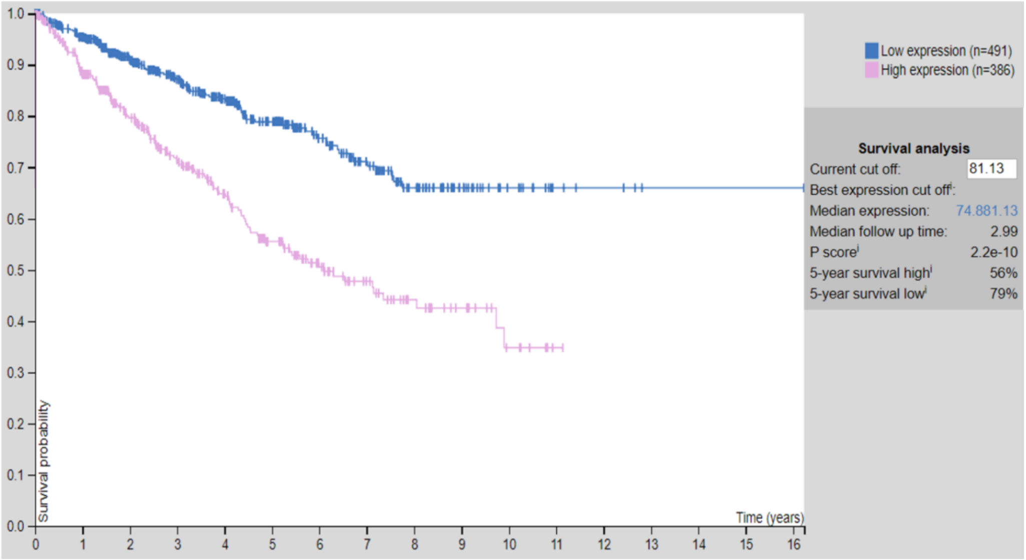

Supplementary Figure 7. The over survival of TIP-B1 in RCC from Human Protein Atlas website

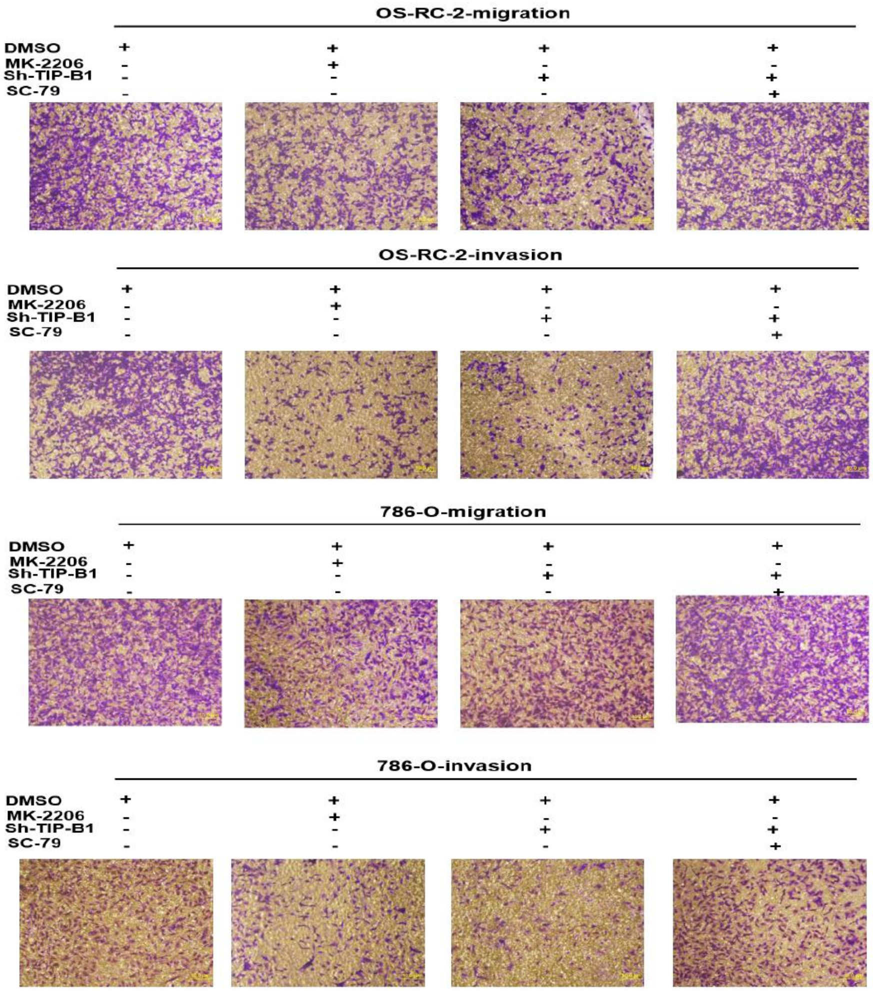

Supplementary Figure 8. The rescue experiments of transwell assay.
