# Supplementary material for: TIP-B1 promotes kidney clear cell carcinoma growth and metastasis via EGFR/AKT signaling
Source: Aging (Albany NY). 2019 Sep 27;11(18):7914–37. doi: 10.18632/aging.102298 (PMC6782011; doi:10.18632/aging.102298)
Supplement: Supplementary Tables 2-5 [file aging-11-102298-s002.pdf]

SUPPLEMENTARY TABLES

Supplementary Table 2. Comparison of baseline clinicopathological characteristics based on TCGA.

	TCGA Cohort(N= 533)			
	Cases No.	TIP-B1		P
		Low	High	
**Age (years)**				
**≤60**	264	161	103	0.795
**>60**	269	167	102	
**Gender**				
**Male**	345	198	147	*0.008*
**Female**	188	130	58	
**Laterality**				
**Left**	251	147	104	0.172
**Right**	281	181	100	
**Bilateral**	1	0	1	
**Pathologic stage**				
**Stage i-ii**	324	238	86	*<0.0001*
**Stage iii-iv**	207	90	117	
**NA**	2			
**Pathology T stage**				
**T1-2**	342	245	97	<0.0001
**T3-4**	191	83	108	
**Pathology N stage**				
**N(-)**	240	146	94	*0.007*
**N(+)**	16	4	12	
**NA**	277			
**Pathology M stage**				
**M(-)**	422	277	145	*<0.0001*
**M(+)**	79	29	50	
**NA**	32			
**Grade**				
**G1-2**	242	183	59	*<0.0001*
**G3-4**	282	137	145	
**NA**	1			
**Cancer Status**				
**Recurrence(-)**	356	253	103	*<0.0001*
**Recurrence(+)**	161	63	98	
**NA**	16			

Supplementary Table 3. Univariate and multivariate Cox proportional hazards analysis of OS from TCGA cohort.

Variables	OS			
	**Univariate analysis**	***P***	**Multivariate analysis**	***P***
	**HR (95%CI)**		**HR (95%CI)**	
**Age (years)**				
**≤60**	1.000	<0.204	NA	
**>60**	1.213(0.900-1.634)			
**TIP-B1**				
**High**	1.000	*<0.0001*	1.000	*0.015*
**Low**	0408(0.302-0.551)		0.669(0.483-0.926)	
**Gender**				
**Male**	1.000	0.737	NA	
**Female**	1.054(0.775-1.434)			
**Pathologic stage**				
**Stage i-ii**	1.000	*<0.0001*	1.000	0.224
**Stage iii-iv**	3.727(2.750-5.050)		1.546(0.766-3.117)	
**Pathology T stage**				
**T1-2**	1.000	*<0.0001*	1.000	0.853
**T3-4**	3.138(2.320-4.245)		1.059(0.580-1.933)	
**Pathology N stage**				
**N(-)**	1.000	*<0.0001*	1.000	0.417
**N(+)**	3.492(1.857-6.565)		1.312(0.682-2.524)	
**NA**	0.829(0.610-1.126)			
**Pathology M stage**				
**M(-)**	1.000	*<0.0001*	1.000	*0.016*
**M(+)**	4.464(3.269-6.095)		1.612 (1.092-2.382)	
**NA**	0.913(0.289-2.889)			
**Grade**				
**G1-2**	1.000	*<0.0001*	1.000	*0.038*
**G3-4**	2.632(1.874-3.696)		1.481(1.021-2.147)	
**NA**	0.750(0.100-5.260)			
**Cancer_Status**				
**Recurrence(-)**	1.000	*<0.0001*	1.000	*<0.0001*
**Recurrence(+)**	4.856(3.528-6.682)		2.763(1.907-4.003)	
**NA**	3.177(1.367-7.384)			

Supplementary Table 4. Sequence of primers for real-time polymerase chain reaction primer.

Primer	Sequence (5’ to 3’)
ZO-1-F	TGCCATTACACGGTCCTCTG
ZO-1-R	GGTTCTGCCTCATCATTTCCTC
E-Cadherin-F	GTAGGAAGGCACAGCCTGTC
E-Cadherin-R	CAGCAAGAGCAGCAGAATCA
N-Cadherin-F	GAGCATGCCAAGTTCCTGAT
N-Cadherin-R	TGGCCACTGTGCTTACTGAA
Vimentin-F	CTGCAGGACTCGGTGGACTT
Vimentin-R	GAAGCGGTCATTCAGCTCCT
Twist-F	GAGTCCGCAGTCTTACGAGG
Twist-R	CTGCCCGTCTGGGAATCACT
Snail-F	ACCACTATGCCGCGCTCTT
Snail-R	GGTCGTAGGGCTGCTGGAA
Slug-F	TGTTGCAGTGAGGGCAAGAA
Slug-R	GACCCTGGTTGCTTCAAGGA
GAPDH-F	ACGGATTTGGTCGTATTGGG
GAPDH-R	TGATTTTGGAGGGATCTCGC

Supplementary Table 5. List of antibodies and reagents used for studies.

Name	Supplier	Catalog
TIP-B1	. Sigma-Aldrich	**HPA030848**
ZO-1	Abcam	ab96587
E-Cadherin	Abcam	ab40772
N-Cadherin	Abcam	ab18203
Vimentin	Abcam	ab92547
Twist	Abcam	ab49254
Snail	Abcam	ab53519
Slug	Abcam	ab183760
Total Akt (T-Akt)	Cell Signaling Technology	#9272
Phosphorylated Akt (p-Akt)	Cell Signaling Technology	#4051
Total ERK(T-ERK)	Cell Signaling Technology	#9102
Phosphorylated ERK(p-ERK)	Cell Signaling Technology	#9101
Total p38(T-p38)	Cell Signaling Technology	#9212
Phosphorylated p38(p-p38),	Cell Signaling Technology	#4511
EGFR	Cell Signaling Technology	#2232
Phosphorylated EGFR(p-EGFR, at Y992,Y1045,Y1068)	Cell Signaling Technology	#2235/#2237/#2234
GAPDH	Abcam	ab9484
MK-2206	Selleck	S1078
SC79	Selleck	S7863
AG-1478	Selleck	S2728
EGF	Sigma-Aldrich	E9644
